# The Fecal Microbiota Is Already Altered in Normoglycemic Individuals Who Go on to Have Type 2 Diabetes

**DOI:** 10.3389/fcimb.2021.598672

**Published:** 2021-02-18

**Authors:** Li Wang, Xinwen Yu, Xiaoqiang Xu, Jie Ming, Zhifeng Wang, Bin Gao, Ying Xing, Jie Zhou, Jianfang Fu, Tao Liu, Xiangyang Liu, Malgorzata A. Garstka, Xiaokai Wang, Qiuhe Ji

**Affiliations:** ^1^Endocrinology Research Center, Xijing Hospital, Fourth Military Medical University, Xi’an, China; ^2^Department of Bioinformatics, Aimigene Institute, Shenzhen, China; ^3^Core Research Laboratory, The Second Affiliated Hospital of Xi’an Jiaotong University, Xi’an, China

**Keywords:** type 2 diabetes, gut microbiota, normoglycemic individuals, *Bifidobacterium*, nested case-control study

## Abstract

**Objective:**

Mounting evidence has suggested a link between gut microbiome characteristics and type 2 diabetes (T2D). To determine whether these alterations occur before the impairment of glucose regulation, we characterize gut microbiota in normoglycemic individuals who go on to develop T2D.

**Methods:**

We designed a nested case-control study, and enrolled individuals with a similar living environment. A total of 341 normoglycemic individuals were followed for 4 years, including 30 who developed T2D, 33 who developed prediabetes, and their matched controls. Fecal samples (developed T2D, developed prediabetes and controls: n=30, 33, and 63, respectively) collected at baseline underwent metagenomics sequencing.

**Results:**

Compared with matched controls, individuals who went on to develop T2D had lower abundances of *Bifidobacterium longum, Coprobacillus unclassified*, and *Veillonella dispar* and higher abundances of *Roseburia hominis, Porphyromonas bennonis*, and *Paraprevotella unclassified*. The abundance of *Bifidobacterium longum* was negatively correlated with follow-up blood glucose levels. Moreover, the microbial Kyoto Encyclopedia of Genes and Genomes (KEGG) pathways of carbohydrate metabolism, methane metabolism, amino acid metabolism, fatty acid metabolism, and membrane transport were changed between the two groups.

**Conclusions:**

We found that fecal microbiota of healthy individuals who go on to develop T2D had already changed when they still were normoglycemic. These alterations of fecal microbiota might provide insights into the development of T2D and a new perspective for identifying individuals at risk of developing T2D.

## Introduction

According to the latest report of the International Diabetes Federation Diabetes Atlas in 2019 (International Diabetes Federation, 2019), there are 463 million diabetes patients worldwide. Type 2 diabetes (T2D) has a high prevalence, severe complications, and causes serious economic losses. Therefore, novel diagnostic markers that could help identify high-risk individuals and new treatment options are needed to improve the prognosis in this population.

The increasing awareness of gut microbiota and its role in host metabolism have promoted a great interest in developing gut microbiota-related diagnostic and therapeutic targets for many diseases. Metagenomics sequencing techniques have dramatically expanded our knowledge of the pathogenesis of T2D. The gut of a healthy human is estimated to be home to around 100 trillion bacteria, roughly an order of magnitude higher than the number of host somatic cells (Goodrich et al., 2014; Schnorr et al., 2014). Mounting evidence has suggested a link between the gut microbiome and diseases (Khan et al., 2014; Peterson et al., 2015), including T2D (Baothman et al., 2016; Sato et al., 2017; Sircana et al., 2018). Although it is likely that the changes in gut microbiota diverge between different populations, patients with T2D were characterized by the similar alterations, a reduction in the abundance of certain common butyrate-producing bacteria and an augmentation in some opportunistic pathogens, which can lead to enhanced inflammatory stress in T2D (Qin et al., 2012; Karlsson et al., 2013). Moreover, antidiabetic medications have been reported to reduce blood glucose by altering the composition and function of gut microbiota. For example, metformin increases short-chain fatty acid (SCFA)-producing bacteria, and SCFAs are known to improve glucose regulation. This increases the abundance of *Escherichia species* (Forslund et al., 2015; Wu et al., 2017), which are known to produce intestinal side effects similar to those seen with metformin. The improvement in the insulin resistance of T2D patients treated with acarbose was closely associated with increased relative abundances of some probiotics in the gut (Gu et al., 2017; Zhang et al., 2017). Furthermore, dietary fiber improved T2D-associated metabolic disorder by regulating gut microbiome (Dewulf et al., 2013; Zhao et al., 2018).

Alterations in gut microbiota have also been found in patients with prediabetes (Allin et al., 2018). A metagenome-wide association study of fecal microbiota in Chinese participants indicated that gut metagenomic markers could differentiate T2D with a high level of specificity (Qin et al., 2012). Whether there is a difference in gut microbiota in normoglycemic individuals before the onset of T2D or prediabetes remains unclear. Could normoglycemic participants develop different glucose metabolism outcomes because of the changes in the composition and function of their fecal microbiota? We hypothesized that individuals who are more likely to go on to develop T2D could be identified from alterations in their gut microbiota.

Our study aimed to explore the characteristics of gut microbiota in normoglycemic individuals who go on to develop T2D. We analyzed the fecal microbiota from participants of a 4-year follow-up survey, and we studied whether the alterations in their gut microbiota occurred earlier than impaired glucose regulation. These alterations could become a promising predictor of future diabetes.

## Methods

### Participants and Study Design

**Figure 1:**
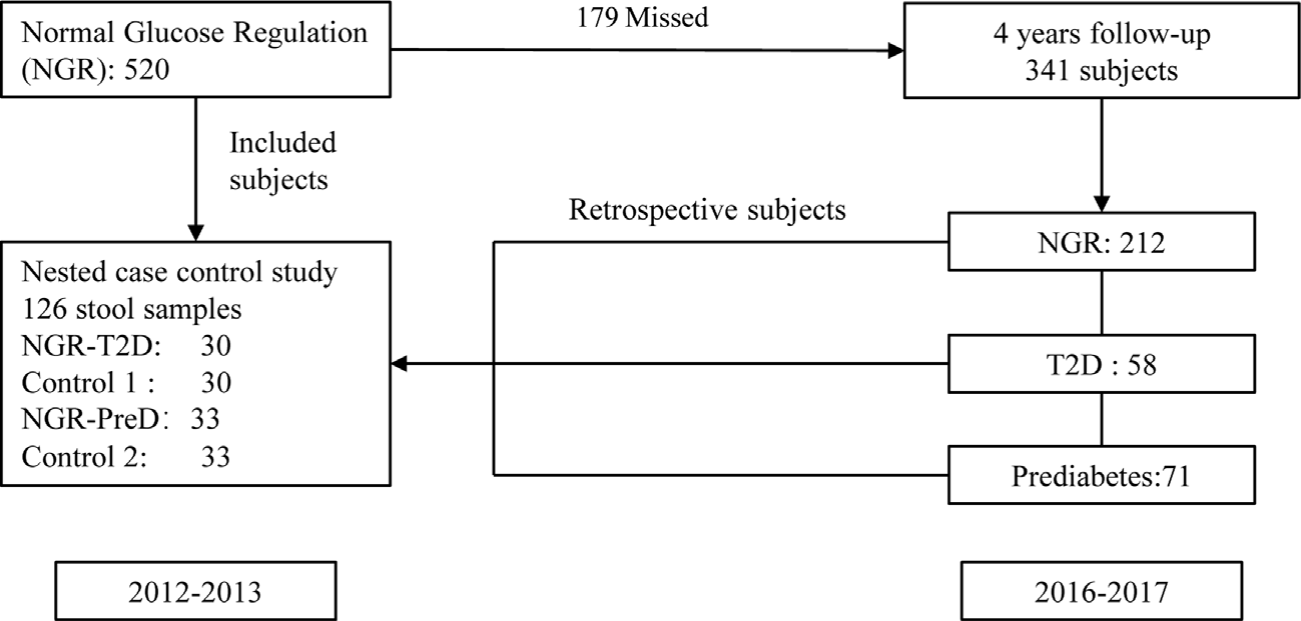
Study design and flow diagram. The diagram presents the design of this nested case-control study. There were 520 individuals with normal glucose regulation (NGR) at baseline (2012–2013), and 341 individuals were followed in 2016–2017. During a 4-year follow-up period, 58 participants developed T2D, and 71 participants developed prediabetes. Participants who had smoking history, taken antibiotics within the past 3 months and other influenced gut microbiota medicines were excluded. Finally, 30 patients with T2D (NGR-T2D) and 33 with prediabetes (NGR-PreD) qualified for metagenomic sequencing. We assigned 30 (control 1) and 33 (control 2) propensity control subjects from the same baseline examination matched with NGR-T2D and NGR-PreD, respectively on age, gender, BMI, FPG, 2h PG, and blood pressure. Qualified: qualified for metagenomic sequencing, including subjects without the use of antibiotics at least 3 months and fecal sample meeting the metagenomic sequencing requirement. All the sequenced stool samples were collected in 2012 and 2013. NGR-T2D: participants had normal glucose regulation at stool collected, and they developed T2D in the follow-up. NGR-PreD: participants had normal glucose regulation at stool collected, and they developed prediabetes in the follow-up. BMI, body mass index; FPG, fasting plasma glucose; 2h PG, plasma glucose 2h after oral glucose tolerance test.

This study was based on a 2007–2008 China National Diabetes and Metabolic Disorders Survey (CNDMDS) (Yang et al., 2010). Our cohort study included 1,915 participants of CNDMDS in Shaanxi province, Northwestern China. In total, 520 participants had normal glucose regulation (NGR) at baseline (2012–2013). Among them, 341 (65.6%) participants were followed in 2016 and 2017. During a 4-year follow-up period, 58 participants developed T2D, and 71 participants developed prediabetes. Participants who had smoking history, taken antibiotics within the past 3 months and other influenced gut microbiota medicines were excluded. Finally, 30 patients with T2D (NGR-T2D) and 33 with prediabetes (NGR-PreD) qualified for metagenomic sequencing. Then, we compared the demographic characteristics between participants with and without fecal samples, but there were no significant differences between them (**Supplemental Table S1**). We matched 30 (control 1) and 33 (control 2) control participants from the same baseline examination for age, gender, body mass index (BMI), fasting plasma glucose (FPG), 2-h postprandial plasma glucose (2h PG), and blood pressure (Wang et al., 2011) by using propensity matching. Briefly, sex-specific, logistic regression models were used to generate the propensity scores. For these models, diabetes or prediabetes was the outcome variable, and the following variables served as covariates: age, BMI, FPG, 2 h PG, and blood pressure. Each case was matched to the control with the closest exam- and gender-specific propensity score, provided the difference in propensity scores was < 0.15 (on a scale of 0.0 to 1.0). Each control was only used once (**Figure 1**). All the sequenced stool samples were collected in 2012 and 2013.

At each visit, standardized questionnaires were used to collect demographic characteristics, personal and family medical history, and lifestyle risk factors. Participants also underwent routine laboratory tests, including a standard 2 h oral glucose tolerance test (OGTT) with 75 g of glucose in solution after at least an 8-h overnight fast. Written informed consent was obtained from each participant before data collection. Institutional review board approvals covered every participant in the study.

### Definition

T2D was defined according to WHO criteria (Gabir et al., 2000) in 1999 as FPG≥126 mg/dl (7.0 mmol/L) or 2 h PG ≥200 mg/dl (11.1 mmol/L) or taking antidiabetic drugs. Prediabetes was considered as FPG ≥110 and <126 mg/dl (≥6.1 and <7.0 mmol/L) or 2 h PG ≥140 and 200 mg/dl (≥7.8 and <11.1 mmol/L).

### Stool Sample Collection and DNA Extraction

Fecal samples freshly collected from each participant were immediately frozen at −20°C, transported to the laboratory in an ice pack and stored at −80°C upon arrival. Bacterial DNA was extracted at Novogene Bioinformatics Technology (Beijing, China) using the sodium dodecyl sulfate (SDS) method. DNA concentration and purity were assessed on 1% agarose gels, and DNA was subsequently diluted to 1 ng/μL using sterile water. DNA degradation degree and potential contamination were monitored on 1% agarose gels. DNA purity (OD260/OD280 and OD260/OD230) was determined using the NanoPhotometer^®^ Spectrophotometer (Implen, CA, USA). DNA concentration was measured using the Qubit^®^ dsDNA Assay Kit in Qubit^®^ 2.0 Fluorometer (Life Technologies, Carlsbad, CA, USA).

### Metagenomic Shotgun Sequencing

All samples were paired-end sequenced on an Illumina HiSeq Platform (insert size 350 bp, read length 151 bp) at Novogene Bioinformatics Technology (Beijing, China). Adapter and low-quality reads were discarded, and the cleaned reads were filtered from human host DNA based on the human genome reference (hg19) as previously described (Qin et al., 2012). We acquired 987.66 Gb of high-quality pair-end reads from 126 human gut microbiome samples with an average of 7.7 Gb per sample.

### Taxonomic and Functional Profiling

Taxonomic profiling of the metagenomic samples was performed using MetaPhlAn2 (v2.7.7) (Truong et al., 2015), which uses a library of clade-specific markers to provide pan-microbial (bacterial, archaeal, viral, and eukaryotic) quantification at the species level. MetaPhlAn2 was run using default settings.

To obtain the functional profile, the high-quality reads were aligned to the updated gut microbiome gene catalog (Li et al., 2014) using SOAP2 (v2.22) with a threshold of more than 90% identity over 95% of the length (Gu et al., 2017). Sequence-based gene abundance profiling was performed as previously described (Li et al., 2014). Briefly,

Step 1: calculation of the copy number of each gene:

$$b_{i}=\frac{x_{i}}{L_{i}}$$

Step 2: calculation of the relative abundance of gene i

$$a_{i}=\frac{b_{i}}{\sum_{j}b_{j}}=\frac{\frac{x_{i}}{L_{i}}}{\sum_{j}\frac{x_{j}}{L_{j}}}$$

*a_i_* : the relative abundance of gene *i* in sample *S*.

*L_i_* : the length of gene *i*.

*x_i_* : the times which gene *i* can be detected in sample *S* (the number of mapped reads).

*b_i_* : the copy number of gene *i* in the sequenced data from sample *S*.”

Next, the relative abundances of KEGG orthologous groups (KOs) were summed up from the relative abundance of their respective genes. Differentially enriched KEGG (Kyoto Encyclopedia of Genes and Genomes) pathway/modules were identified according to their reporter score (Patil and Nielsen, 2005; Feng et al., 2015) from the Z-scores of individual KOs. The pathway/modules are determined based on different KOs abundances which could include both KOs that are more or less abundant in the same pathway/modules. A one-tail Wilcoxon rank-sum test was performed on all the KOs that occurred in more than five samples and adjusted for multiple testing using the Benjamin-Hochberg procedure. The Z-score for each KO was then calculated:

$$Z_{KO_{i}}=\theta^{-1}\left(1-P_{KO_{i}}\right),$$

where *θ*^–1^ was the inverse normal cumulative distribution, and $P_{KO_{i}}$as the adjusted *P* value for that KO. The aggregated Z-score for a KEGG pathway (or module) was then:

$$Z_{pathway}=\frac{1}{\sqrt{k}}\sum^{}Z_{KO_{i}}$$

where k is the number of KOs involved in the pathway (or module).

We corrected the background distribution of *Z_pathway_* by subtracting the mean (*µ_k_*) and dividing by the s.d. (*σ_k_*) of the aggregated Z-scores of 1,000 sets of k KO:

$Z_{adjustpathway}=\frac{Z_{pathway}-\mu_{k}}{\sigma_{k}}$ The *Z_adjustedpathway_* was used as the final reporter score for evaluating the enrichment of specific pathways or modules. A reporter score of ≥1.96 (95% confidence according to normal distribution) could be used as a detection threshold for significantly differentiating pathways.

To compare the differences in functional alterations in gut microbiota between our retrospective cohort and T2D case-control cohort, we re-analyzed a published study that used fecal shotgun metagenomics to characterize Chinese T2D patients compared to healthy controls (Qin et al., 2012). To reduce the cohort difference, we selected 120 individuals, 60 T2D patients and 60 controls, from the original study. These were matched for age, gender, BMI, and blood pressure. The data processing of the T2D cohort was consistent with the present study.

### Statistical Analysis

Unless otherwise stated, statistical analyses were made in the R (v4.0.0) software. Differential abundance of phyla, genera, and species were tested by two-tailed Wilcoxon rank-sum test, and *P* < 0.05 was considered significantly different. Spearman’s rank-order correlation was used to determine the strength and direction of the monotonic relationships between two variables. The corresponding correlation network was visualized using the Cytoscape (v3.72) software program. Other plots were visualized with ggplot2 (v3.30). Results were not adjusted for multiple testing.

## Results

### Participant Characteristics

All study participants were residents of Xi’an, Shaanxi province in China and had similar living conditions and dietary habits. One hundred twenty-six normoglycemic individuals were enrolled in the study including 30 who developed T2D (NGR-T2D) and 33 who developed prediabetes (NGR-PreD) at 4-years follow-up and their matched controls (control 1, n = 30; control 2, n = 33). The main clinical and biochemical characteristics of the included participants are shown by case-control status in **Table 1**.

**Table 1:** Demographic characteristics of cases and controls.

	NGR-T2D	Control 1	*P*^#^ value	NGR-PreD	Control 2	*P*^#^ value
	n = 30	n = 30		n = 33	n = 33	
Age (years)	56.1± 7.93	52.6 ± 8.81	0.115	58.7 ± 11.25	57.1 ± 10.70	0.573
Gender (male:female)	12:18	12:18	1.000	11:22	11:22	1.000
Height (cm)	160 ± 7.6	162 ± 9.0	0.227	161 ± 8.9	160 ± 7.2	0.858
Weight (kg)	64.5 ± 10.22	66.3 ± 11.00	0.528	62.6 ± 12.14	63.7 ± 8.84	0.671
BMI (kg/m2)	25.2 ± 3.05	25.1 ± 3.32	0.870	24.1 ± 3.17	24.8 ± 3.29	0.383
Waist (cm)	87.6 ± 8.14	83.6 ± 11.15	0.125	88.2 ± 11.60	82.1 ± 7.77	0.015
Smoking history (yes:no)	5:16	8:18	0.746	6:18	6:21	1.000
Hypertension (n)	7	5	0.748	2	5	0.258
FPG (mmol/L)	3.96 ± 0.787	4.15 ± 0.742	0.362	4.19 ± 0.763	4.02 ± 0.812	0.386
2h PG (mmol/L)	5.61 ± 1.020	5.44 ± 1.050	0.537	5.45 ± 1.240	5.55 ± 1.250	0.747
TC, mmol/L	4.73 ± 1.093	4.40 ± 0.925	0.220	4.44 ± 0.874	4.39 ± 0.822	0.816
TG, mmol/L	2.12 ± 1.886	1.48 ± 0.717	0.094	1.54 ± 1.077	1.40 ± 0.754	0.562
HDL-C, mmol/L	1.24 ± 0.302	1.19 ± 0.296	0.512	1.28 ± 0.292	1.26 ± 0.255	0.707
LDL-C, mmol/L	2.79 ± 0.901	2.67 ± 0.787	0.573	2.65 ± 0.894	2.65 ± 0.707	0.984
ALT, U/L	22.5 ± 8.77	22.8 ± 11.95	0.913	23.7 ± 15.45	28.0 ± 31.38	0.485
AST, U/L	21.7± 4.34	19.8 ± 4.37	0.098	22.8 ± 8.72	24.7 ± 12.11	0.473
UA, mmol/L	227 ± 78.7	246 ± 66.9	0.332	220 ± 71.8	219 ± 70.8	0.968
Follow-FPG (mmol/L)	8.12 ± 2.815	4.88 ± 0.630	<0.001	5.19 ± 0.699	4.89 ± 0.588	0.064
Follow-2h PG (mmol/L)	15.78 ± 4.270	5.78 ± 1.003	<0.001	9.27 ± 0.931	6.08 ± 1.056	<0.001
Follow-TC, mmol/L	5.40 ± 1.305	4.99 ± 0.943	0.171	4.89 ± 1.049	5.00 ± 0.831	0.630
Follow-TG, mmol/L	1.99 ± 0.966	1.48 ± 0.653	0.022*	1.76 ± 0.954	1.25 ± 0.695	0.015
Follow-HDL-C, mmol/L	1.24 ± 0.225	1.27 ± 0.254	0.626	1.30 ± 0.309	1.43 ± 0.324	0.106
Follow-LDL-C, mmol/L	3.56 ± 1.194	3.14 ± 0.588	0.096	3.03 ± 0.900	3.10 ± 0.698	0.725
Follow-ALT, U/L	31.5 ± 17.60	28.4 ± 16.50	0.480	31.2 ± 28.00	38.7 ± 62.40	0.536
Follow-AST, U/L	24.5 ± 8.47	24.5 ± 6.60	0.986	25.6 ± 11.66	30.5 ± 29.81	0.383
Follow-UA, mmol/L	5.96 ± 2.059	4.98 ± 0.976	0.139	6.24 ± 1.732	6.09 ± 1.594	0.795
Family history of diabetes (n)	13	3	0.007	4	2	0.672
Family history of hypertension (n)	16	12	0.438	18	14	0.460
Family history of hyperlipemia (n)	5	3	0.706	6	5	1.000
Family history of hyperuricemia or gout (n)	1	–	1.000	–	–	1.000
Family history of CHD (n)	7	5	0.748	3	3	1.000
Family history of myocardial infarction (n)	6	3	0.472	1	1	1.000
Family history of stroke (n)	3	5	0.706	3	5	0.475
Family history of heart failure (n)	1	–	0.492	–	–	1.000

Data are expressed as mean ± SD or n. Student’s t test for continuous variables, Mann–Whitney U test for abnormally distributed data, and chi-square test for categorical variables.

P^#^: NGR-T2D vs. control 1; P^##^: NGR-PreD vs. control 2.

NGR-T2D: participants had normal glucose regulation at stool collected, and they developed T2D in the follow-up.

NGR-PreD: participants had normal glucose regulation at stool collected, and they developed prediabetes in the follow-up.

BMI, body mass index; FPG, fasting plasma glucose; 2h PG, plasma glucose 2h after oral glucose tolerance test; TC, total cholesterol; UA, uric acid; follow-, following up for 4 years.

### Changes in Gut Microbiota of Normoglycemic Participants Who Went on to Develop T2D

**Figure 2:**
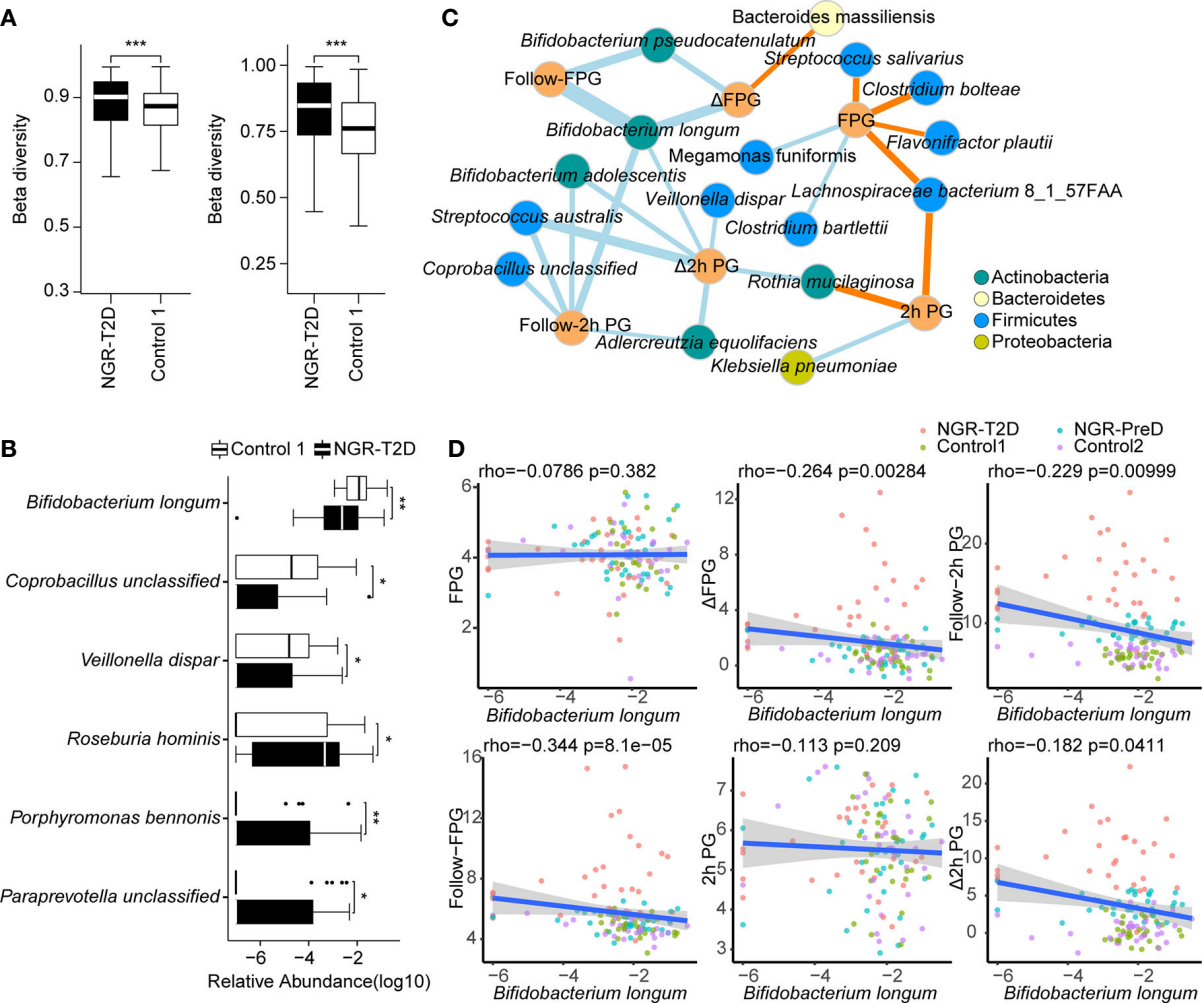
Differences in the fecal microbial communities and the correlation between fecal microbiota and glucose levels. **(A)** The comparison of beta diversity at the gene (left) and species (right) level. **(B)** Species with significantly different abundance in NGR-T2D (n=30) compared with control 1 (n=30). Two-tailed Wilcoxon rank-sum test was used to determine statistical significance, *P < 0.05, **P < 0.01, ***P < 0.001. **(C)** The network of correlations between species and phenotypes from all the individuals (n=126) was established by Spearman’s correlation analysis. Blue edges, represent significantly negative correlations, *P* < 0.05; yellow edges, represent significantly positive correlations, *P* < 0.05. Sizes of the edges represent the |rho| of Spearman’s correlation coefficient. **(D)** Scatter plot of glucose levels *versus* the relative abundance of *Bifidobacterium longum*. The linear regression line and the 95% confidence interval are visualized. The correlations between the *Bifidobacterium longum* abundance and the glucose level were calculated by Spearman’s correlation analysis (n=126). NGR-T2D: participants had normal glucose regulation at stool collected, and they developed T2D in the follow-up. FPG, baseline fasting plasma glucose; 2h PG, baseline plasma glucose 2h after oral glucose tolerance test; follow-FPG, follow-up fasting plasma glucose; follow-2h PG, follow-up 2h postprandial plasma glucose; Δ FPG, the difference between Follow-FPG and FPG; Δ 2h PG, the difference between follow-2h PG and 2h PG.

We carried out metagenomics sequencing on stool samples from normoglycemic individuals collected at baseline. Diversity analyses revealed no significant differences in alpha-diversity of gut microbiota between cases and matched controls (**Supplemental Figures S1** and **S2A, B**). However, higher beta-diversity was observed in the NGR-T2D group than in the control 1 group, including at the gene (*P* = 3.0e−6) and species (*P* = 2.2e−13) level (**Figure 2A**). Beta-diversity was not significantly different between NGR-PreD and control 2 groups (**Supplemental Figure S2C**), which indicated a more heterogeneous microbial community structure in individuals who went on to develop T2D.

After filtering out species with a low-occurrence (i.e., present in fewer than 30% individuals), we compared species abundance in cases and matched controls and found that gut microbiota of NGR-T2D and NGR-PreD groups was different from the corresponding controls 4 years before diagnosis of T2D and prediabetes, respectively (**Figure 2B**). Differential species tests showed decreased abundances of *Bifidobacterium longum*, *Coprobacillus unclassified*, and *Veillonella dispar*, along with increased abundances of *Roseburia hominis, Porphyromonas bennonis*, and *Paraprevotella unclassified* in the NGR-T2D group compared with the Control 1 group. Although these changes were not observed in the NGR-PreD group compared with the control 2 group, the abundance of *Klebsiella oxytoca* was significantly lower in NGR-PreD group (**Supplemental Figure S2D**). These observations suggest that the specific species in gut microbiota may correlate with different outcomes of host glucose regulation.

### Correlation Between Fecal Microbiota and Glucose Level

We further illustrated the correlation between the gut microbiota and T2D by exploring correlations between the microbiota and glucose levels (**Figure 2C**). In total, 25 pairs of significant and robust relationships (edges) were identified from 22 parameters (nodes) (*P* < 0.05), including 16 species and six indicators of glucose level. We identified 7 positive and 18 negative correlations using Spearman’s correlation coefficient. Most species correlated with glucose levels belonged to *Actinobacteria* and *Firmicutes*. Among all tested species, three with a reduced abundance in the NGR-T2D group, as demonstrated in **Figure 2B**, showed negative correlations with glucose levels (**Figure 2C**). Especially interesting was *Bifidobacterium longum* (**Figure 2D**). The abundance of *Bifidobacterium longum* negatively correlated with follow-FPG (FPG at 4-year follow-up) (Spearman’s correlation, r: −0.344, *P* < 0.001), Follow-2 h PG (2 h PG at 4-year follow-up) (Spearman’s correlation, r: −0.229, *P* < 0.01), and the difference in FPG and 2h PG between 4-year follow up and baseline (Spearman’s correlation, r: −0.264, *P <*0.01 and r: −0.182, *P <*0.05, respectively). Another two species, *Coprobacillus unclassified* and *Veillonella dispar*, negatively correlated with follow-2h PG and the difference in FPG and 2h PG, respectively. However, changes in microbiota species abundances that would be correlated to future glucose regulation requires additional study. We also observed that *Paraprevotella unclassified* and *Veillonella dispar* correlated with Follow-uric acid and HDL-C, respectively, and *Coprobacillus unclassified* was correlated with alanine transaminase and aspartate transaminase. In addition, the abundance of certain species, including *Parabacteroides distasonis*, *Rothia mucilaginosa*, and *Bacteroides plebeius*, correlated with other metabolic indicators including lipids, uric acid, and aminotransferases (**Supplemental Figure S3**).

### Functional Characterization of Gut Microbiota in NGR Participants Before the Onset of T2D

**Figure 3:**
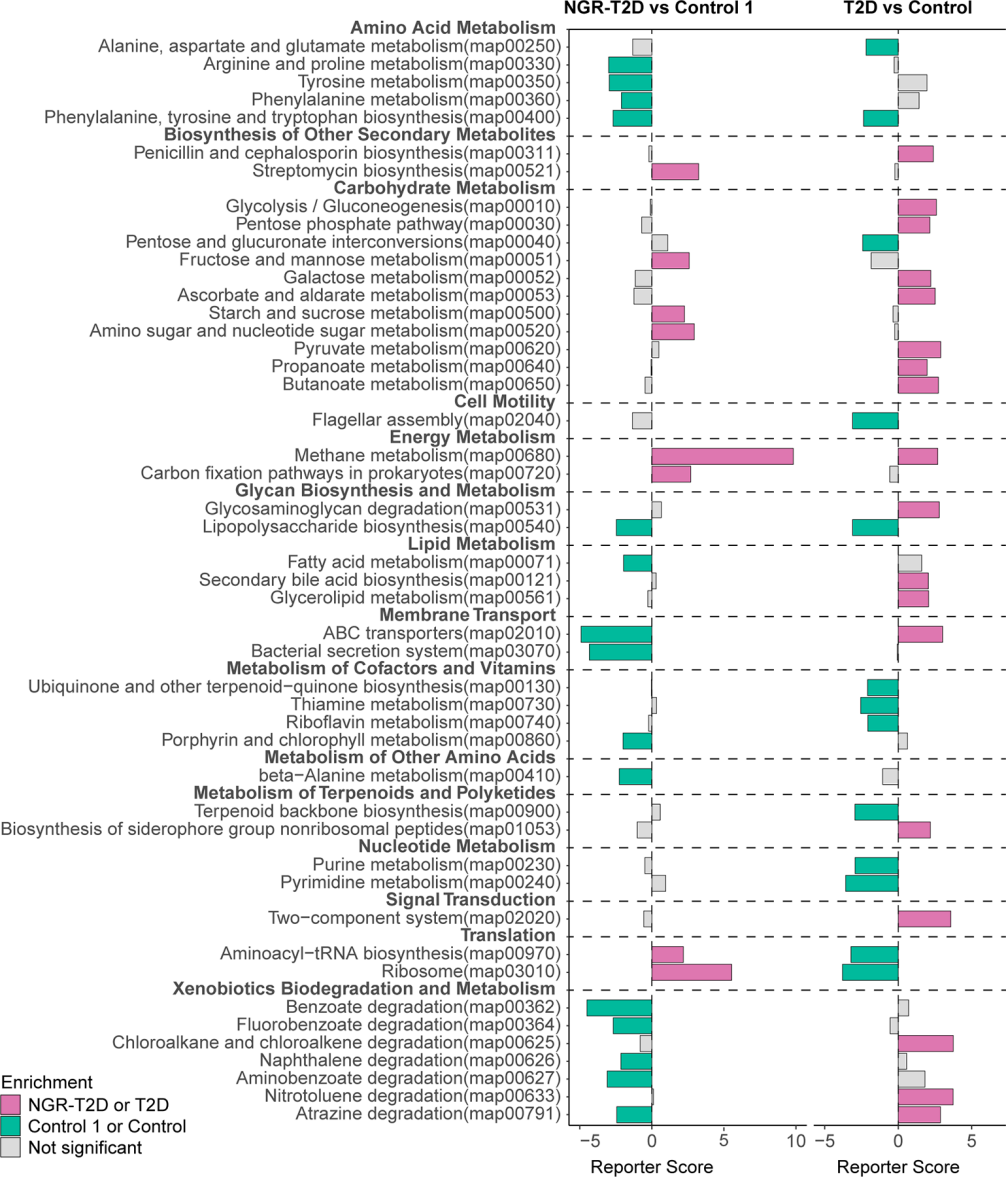
Differential enrichment of functional pathways. Left: differential enrichment between NGR-T2D (n = 30) and control 1 (n = 30) group. Right: differential enrichment between Chinese T2D patients (n = 60) and healthy controls (n = 60). X-axis represents reporter score. NGR-T2D: participants had normal glucose regulation at stool collected, and they developed T2D in the follow-up.

Numerous metabolites produced by the gut microbiota can influence our metabolism (Cani, 2018). To determine the functional capacity of gut microbiota in the healthy participants and patients who would go on to develop T2D, we analyzed fecal microbiota of study participants using KOs. We found that gut microbiota of NGR-T2D group was enriched in the pathways of carbohydrate metabolism (including fructose and mannose metabolism; starch and sucrose metabolism; amino sugar and nucleotide sugar metabolism) and methane metabolism. The gut microbiota of control 1 group was enriched in pathways of amino acid metabolism (including arginine, proline, tyrosine and phenylalanine metabolism; phenylalanine, tyrosine and tryptophan biosynthesis), lipopolysaccharide biosynthesis, fatty acid metabolism, membrane transport (including bacterial secretion system and ABC transporters), xenobiotics biodegradation, and metabolism (**Figure 3**, left and **Supplemental Table S2**).

To further explore the functional alterations in gut microbiota, we screened 120 individuals, including T2D patients and healthy controls from another Chinese study matched on age, gender BMI, and blood pressure (**Figure 3**, right) (Qin et al., 2012). Most functional pathways were differently enriched. However, the changes in two functional microbial pathways were consistent between T2D patients enrolled in the study by Qin et al. (2012) and normoglycemic individuals who later went on to develop T2D enrolled in this study: phenylalanine, tyrosine, and tryptophan biosynthesis and methane metabolism. These pathway differences may provide new insights into the functional changes in gut microbiota prior to impaired glucose regulation.

## Discussion

Here, we report the first retrospective study that investigated the changes in fecal microbiota in normoglycemic individuals before they developed T2D. Participants were inhabitants of the city of Xi’an in China who had a similar living environment. We analyzed the gut microbiota of participants with NGR at baseline and found that fecal microbiota of participants who went on to develop T2D had shown significant alterations at least 4 years before the onset of impaired glucose regulation.

Previous studies have demonstrated a moderate degree of gut microbial dysbiosis in T2D (Qin et al., 2012). Individuals with prediabetes also had aberrant fecal microbiota (Forslund et al., 2015; Wu et al., 2017; Allin et al., 2018). Moreover, antidiabetic medications and diets could regulate the composition and function of gut microbiota through specific pathways (Dewulf et al., 2013; Forslund et al., 2015; Kovatcheva-Datchary et al., 2015; Wu et al., 2017; Gu et al., 2017; Zhang et al., 2017). Nonetheless, these studies, conducted in already diabetic patients, focused on the association between diabetes and gut microbiota and emphasized that altered microbiota could directly regulate glucose regulation in the host.

In this study, we compared gut microbiota in normoglycemic individuals and followed them after 4 years and demonstrated that the gut microbiota had already changed in the healthy individuals before they developed T2D. These individuals showed a significantly decreased abundance of *Bifidobacterium longum*, *Coprobacillus unclassified*, and *Veillonella dispar*, and increased the abundance of *Roseburia hominis, Porphyromonas bennonis*, and *Paraprevotella unclassified*. Among these, *Bifidobacterium* is the most studied species as a probiotic. Previous studies have reported that *Bifidobacterium* abundance was decreased in T2D participants (Sedighi et al., 2017; Gonai et al., 2017; Sroka-Oleksiak et al., 2020). Metformin increased the relative abundance of *Bifidobacterium adolescentis* (Rodriguez et al., 2018), and acarbose increased the gut content of *Bifidobacterium longum* in T2D patients (Su et al., 2015). In addition, *Roseburia hominis*, a butyrate-producing bacteria, has recently been shown to exert the immunomodulatory properties in gut inflammation and to show significantly reduced levels in the guts of patients with ulcerative colitis (Machiels et al., 2014; Patterson et al., 2017).

We found that in the normoglycemic individuals, only the abundance of *Bifidobacterium longum* at baseline correlated negatively with the glucose level at the follow-up for both FPG and 2h PG, which suggests that healthy individuals with depleted *Bifidobacterium* species may have a higher risk of developing T2D later in life. Another study also showed a significantly lower number of bacteria of the genus *Bifidobacterium* of the duodenal mucosa microbiota in T2D patients, and the numbers of *Bifidobacterium* were positively correlated with HDL-C level, which indicated the potential of the genus *Bifidobacterium* as a biomarker in the progress of T2D (Sroka-Oleksiak et al., 2020). Moreover, oral administration of *Bifidobacterium* species could ameliorate insulin resistance and improve glucose tolerance in mice on a high-fat diet (Kikuchi et al., 2018) and obese diabetes model mice (Ben Othman and Sakamoto, 2020). This shows *Bifidobacterium* species may have an essential role in preventing the development of T2D. The pathways of lipopolysaccharide biosynthesis, ABC transporters, arginine, and proline metabolism were decreased in individuals who went on to develop T2D. These pathways were enriched in T2D patients after metformin treatment and *B. adolescentis* increased (Wu et al., 2017). However, there were not any studies to investigate the prevention of T2D by *Bifidobacteria* in humans. Gut-microbial dysbiosis could be detected years before the onset of T2D, suggesting that the composition and diversity of gut microbiota may play a role in the etiology of diabetes in humans.

Furthermore, we analyzed the potential functional roles of the gut microbiota in normoglycemic individuals who progressed to T2D within 4 years. Although our participants were healthy, the gut microbiota from participants who developed T2D was already changed compared to their corresponding controls, as indicated by a difference in abundance of various species and functional microbial pathways. Some of these alterations were consistent with findings from T2D patients, e.g., enrichment of genes involved in methanogenesis (Deepinder et al., 2011; Cesario et al., 2014; Mathur et al., 2016).

Interactions between the gut microbiota, diet, and host genetics may potentially be the initiating factors in the development of abnormal glucose metabolism. A significant number of studies have suggested that metabolites produced by gut microbial species might play an important role in impaired glucose regulation, including modulation of insulin sensitivity. Amino acid fermentation leads to the production of ammonia, amines, H_2_S, phenol, and phenolic derivatives (indole and p-cresol) and was found to affect insulin sensitivity (Khan et al., 2014). Imidazole propionate, a microbial histidine-derived metabolite, was demonstrated to impair insulin signaling at the level of the insulin receptor substrate and subsequently activate the mechanistic target of rapamycin complex 1 (mTORC1) (Koh et al., 2018). Moreover, *Bifidobacterium* has been associated with the production of many potentially health-promoting metabolites including SCFAs, conjugated linoleic acid, and bacteriocins (Arboleya et al., 2016). The TEDDY study indicated that the gut microbial taxonomy, including *Bifidobacterium longum*, is associated with the development of islet autoimmunity or type 1 diabetes in early life (Stewart et al., 2018). Our study also revealed that the difference in microbiota composition of normoglycemic individuals was related to the onset of future T2D. Accordingly, we hypothesize that supplementation with *Bifidobacterium* might prevent the onset of T2D in high-risk individuals. This hypothesis requires animal and clinical validation to elucidate specific mechanisms and to translate into preventive options for T2D.

Microbial markers and metabolites in the blood might predict the risk of diabetes development. A cohort study showed that 16S rDNA gene content in blood was an independent marker of the diabetes risk, and identified microbiota was mostly composed of the *Proteobacteria phylum*, especially *Ralstonia* (Amar et al., 2011). Another cohort study followed for 12 years suggested that branched-chain amino acids (BCAAs) and aromatic amino acids in serum could predict future diabetes (Wang et al., 2011). A subsequent investigation found that increased levels of BCAAs in serum correlated with the abundance of *Proteobacteria copri* in the gut (Pedersen et al., 2016). Additionally, Li et al. explored the gut microbiota-based classifiers to identify individuals with a high risk for T2D in patients from northern china (Li et al., 2020). These studies indicated the potential of microbiota and metabolites in T2D prediction.

We found that the FPG increased significantly in control groups (*P* < 0.0001 for both control 1 and control 2 group). In the other cohort study, compared with subjects with FPG levels less than 85 mg/dl (4.72 mmol/L), those in the 85 to 89 mg/dl (4.72 to 4.94 mmol/L) category were not at significantly greater risk of diabetes (Nichols et al., 2008). Although glucose levels of control groups at follow-up were higher compared to baseline, the risk of diabetes may not increase significantly.

Nonetheless, some limitations of this study should be acknowledged. First, only 126 fecal samples were analyzed by metagenomic sequencing, and known diabetes risk factors include ethnicity, geographical and environmental factors, and dietary habits, which affect the characteristics of gut microbiota. Therefore, a better epidemiological study program should be established to observe the dynamic changes of gut microbiota in individuals who may go on to have T2D before and during the development of glucose metabolism disorders and so validate the feasibility of the predictive model of the influence of the gut microbiota on diabetes. Certainly, changes in gut microbiota of individuals may be different due to different living conditions and dietary structure, so it is necessary to determine whether this phenomenon persists across geographic regions or not. Our study provides a theoretical basis for the next multicenter study. Another limitation is that the follow-up rate of normoglycemic individuals was below 70%, which is a common rate in China. Finally, because all participants in this study were Chinese men and women, the applicability of our findings to other ethnic groups requires validation.

In conclusion, our findings provide a novel perspective of the association between gut microbiota and T2D. The fecal microbiota of healthy individuals who go on to develop T2D have already changed by the time they cease to be normoglycemic, suggesting microbiota changes may be used to identify individuals at high risk for T2D.

## Data Availability Statement

Metagenomic sequencing data for all samples is uploaded to the European Bioinformatic Institute (EBI, https://www.ebi.ac.uk/) database under accession code PRJEB30611. Other data are available from the corresponding author upon reasonable request.

## Ethics Statement

The studies involving human participants were reviewed and approved by Independent Ethics Committee of Xijing Hospital, Fourth Military Medical University. The patients/participants provided their written informed consent to participate in this study.

## Author Contributions

QJ and XW conceived and designed the study. LW, XX, XY, JM, and ZW contributed to the data extraction, performed the analysis, and interpreted the results. LW, JM, and YX wrote the first draft. JM, LW, BG, YX, JZ, JF, TL, and XL contributed to the data collection. QJ, XW, and MG were responsible for critical revision of the manuscript for important intellectual content. All authors contributed to the article and approved the submitted version.

## Funding

This work was supported by grants from the National Natural Science Foundation of China (Grant No.31571415), the Key Research and Development Program of Shaanxi Province, China (Grant No. 2017ZDCXLSF0201) and the National Key Research and Development Program of China (Grant No. 2017YFC1309803). The study funders were not involved in the study design, data collection, analysis, interpretation, or writing of the report.

## Conflict of Interest

The authors declare that the research was conducted in the absence of any commercial or financial relationships that could be construed as a potential conflict of interest.

## Supplementary Material

Supplementary Figure S1 Comparison of microbial alpha diversity between NGR-T2D (n = 30) and Control 1 (n = 30) groups. The comparison of alpha diversity (richness and Shannon’s index) at the gene **(A)** and species **(B)** level. All plotted boxes are interquartile ranges. The center line denotes the median, the boxes cover the 25th and 75th percentiles, and the whiskers extend to the most extreme data point, which is no more than 1.5 times the length of the box away from the box. Points outside the whiskers represent outlier samples.

Supplementary Figure S2 Comparison of fecal microbial diversity between NGR-PreD (n = 33) and Control 2 (n = 33). The comparison of alpha diversity (richness and Shannon’s index) at the gene **(A)** and species **(B)** level, as well as beta diversity **(C)** at the gene (left) and species (right) level. **(D)** Species with significantly different abundance in NGR-PreD compared with Control 2 and species in **Figure 2B**. All plotted boxes are interquartile ranges. The center line denotes the median, the boxes cover the 25th and 75th percentiles, and the whiskers extend to the most extreme data point, which is no more than 1.5 times the length of the box away from the box. Points outside the whiskers represent outlier samples. Two-tailed Wilcoxon rank-sum test was used to determine statistical significance, *P < 0.05.

Supplementary Figure S3 Correlation between fecal microbiota and clinical indices (n = 126). Heatmap and hierarchical clustering of Spearman’s correlation coefficients between species and clinical indices at baseline and follow up. TC, total cholesterol, TG, triglyceride, HDL-C: high-density lipoprotein cholesterol; LDL-C: low-density lipoprotein cholesterol, UA, uric acid, AST, aspartate transaminase, ALT, alanine transaminase; ^+^P < 0.05; *P < 0.01.
