# Supplementary figures and images for: The Fecal Microbiota Is Already Altered in Normoglycemic Individuals Who Go on to Have Type 2 Diabetes
Source: Front Cell Infect Microbiol. 2021 Feb 18;11:598672. doi: 10.3389/fcimb.2021.598672 (PMC7930378; doi:10.3389/fcimb.2021.598672)

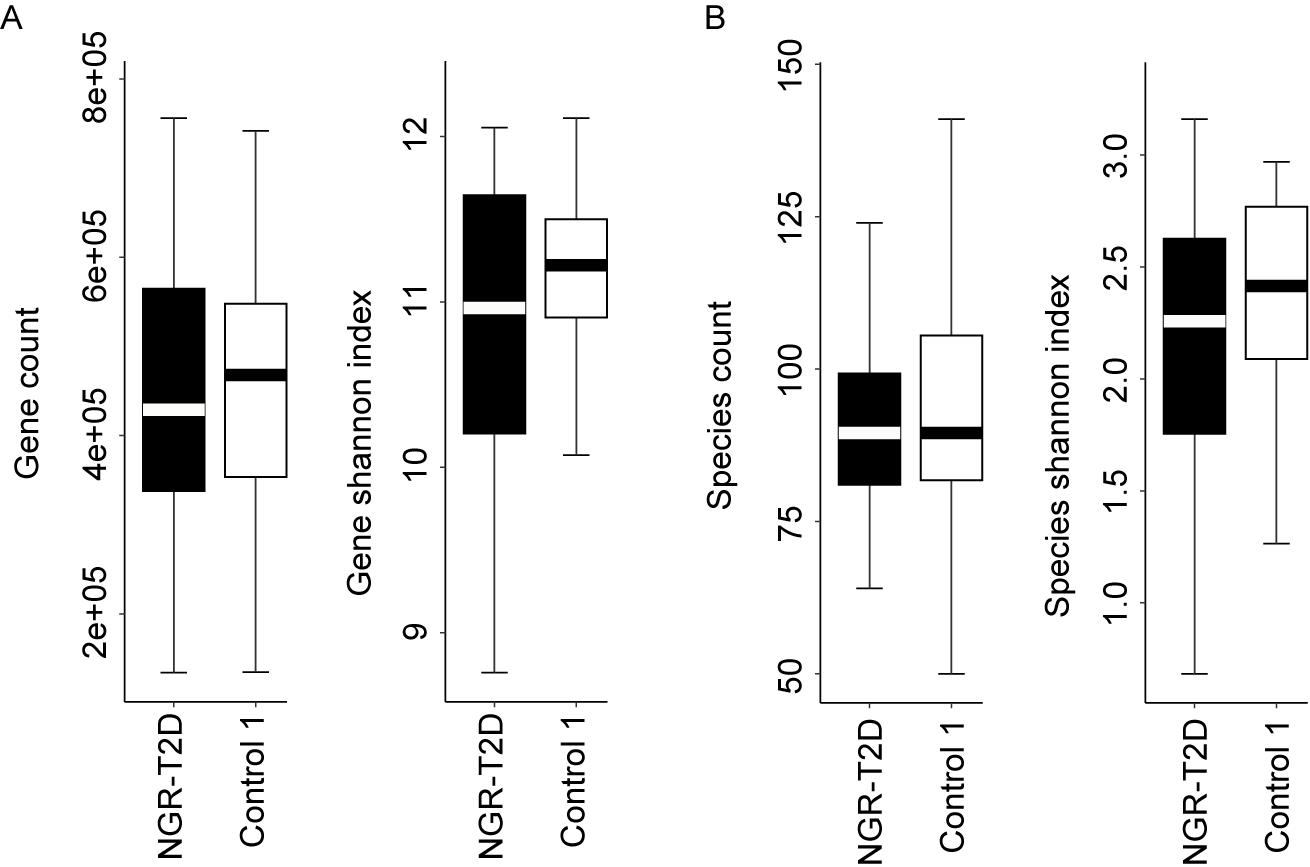

Supplement: Supplementary Figure S1 — Comparison of microbial alpha diversity between NGR-T2D (n = 30) and Control 1 (n = 30) groups. The comparison of alpha diversity (richness and Shannon’s index) at the gene (A) and species (B) level. All plotted boxes are interquartile ranges. The center line denotes the median, the boxes cover the 25th and 75th percentiles, and the whiskers extend to the most extreme data point, which is no more than 1.5 times the length of the box away from the box. Points outside the whiskers represent outlier samples. [file Image_1.tif]

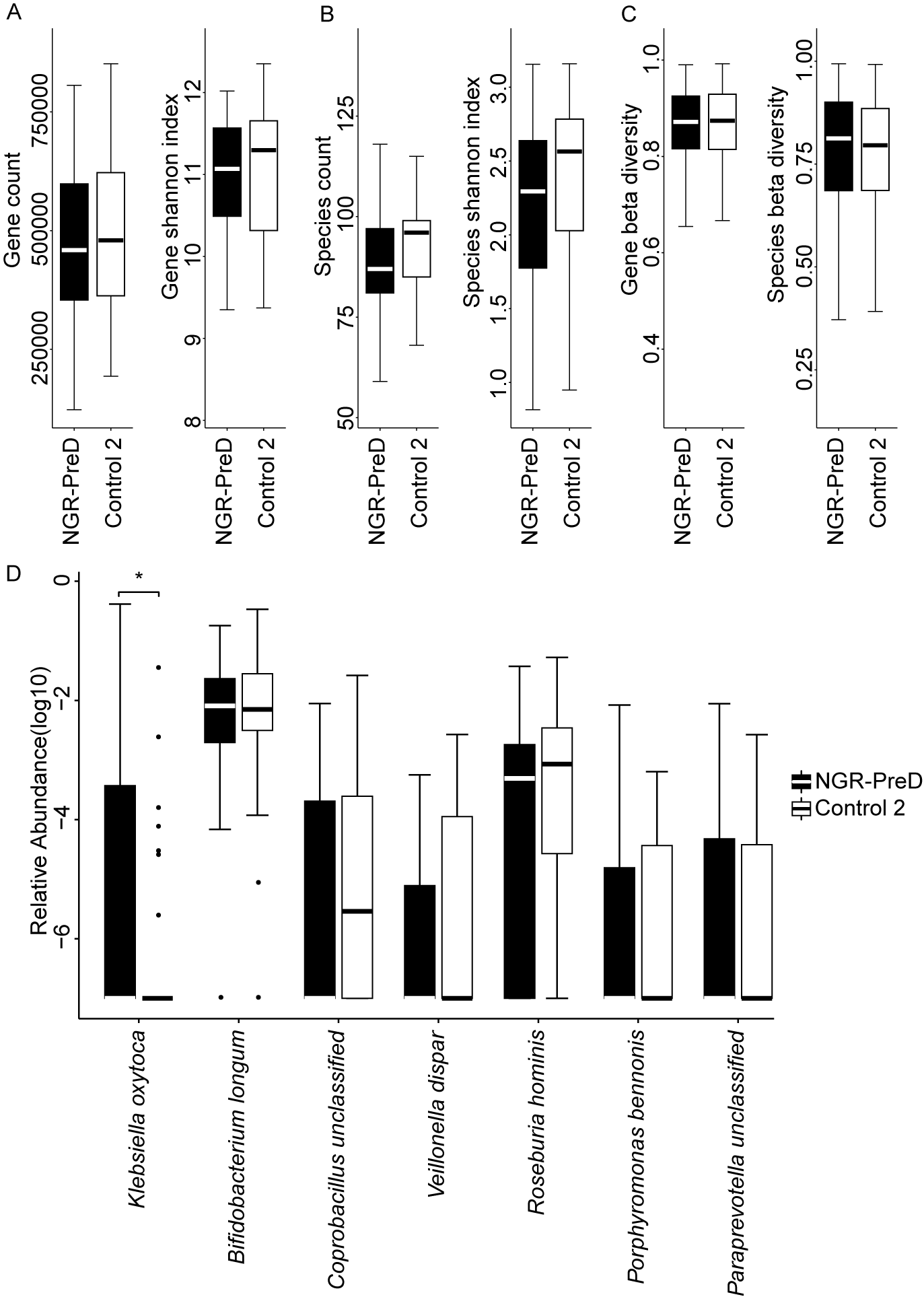

Supplement: Supplementary Figure S2 — Comparison of fecal microbial diversity between NGR-PreD (n = 33) and Control 2 (n = 33). The comparison of alpha diversity (richness and Shannon’s index) at the gene (A) and species (B) level, as well as beta diversity (C) at the gene (left) and species (right) level. (D) Species with significantly different abundance in NGR-PreD compared with Control 2 and species in Figure 2B. All plotted boxes are interquartile ranges. The center line denotes the median, the boxes cover the 25th and 75th percentiles, and the whiskers extend to the most extreme data point, which is no more than 1.5 times the length of the box away from the box. Points outside the whiskers represent outlier samples. Two-tailed Wilcoxon rank-sum test was used to determine statistical significance, *P < 0.05. [file Image_2.tif]

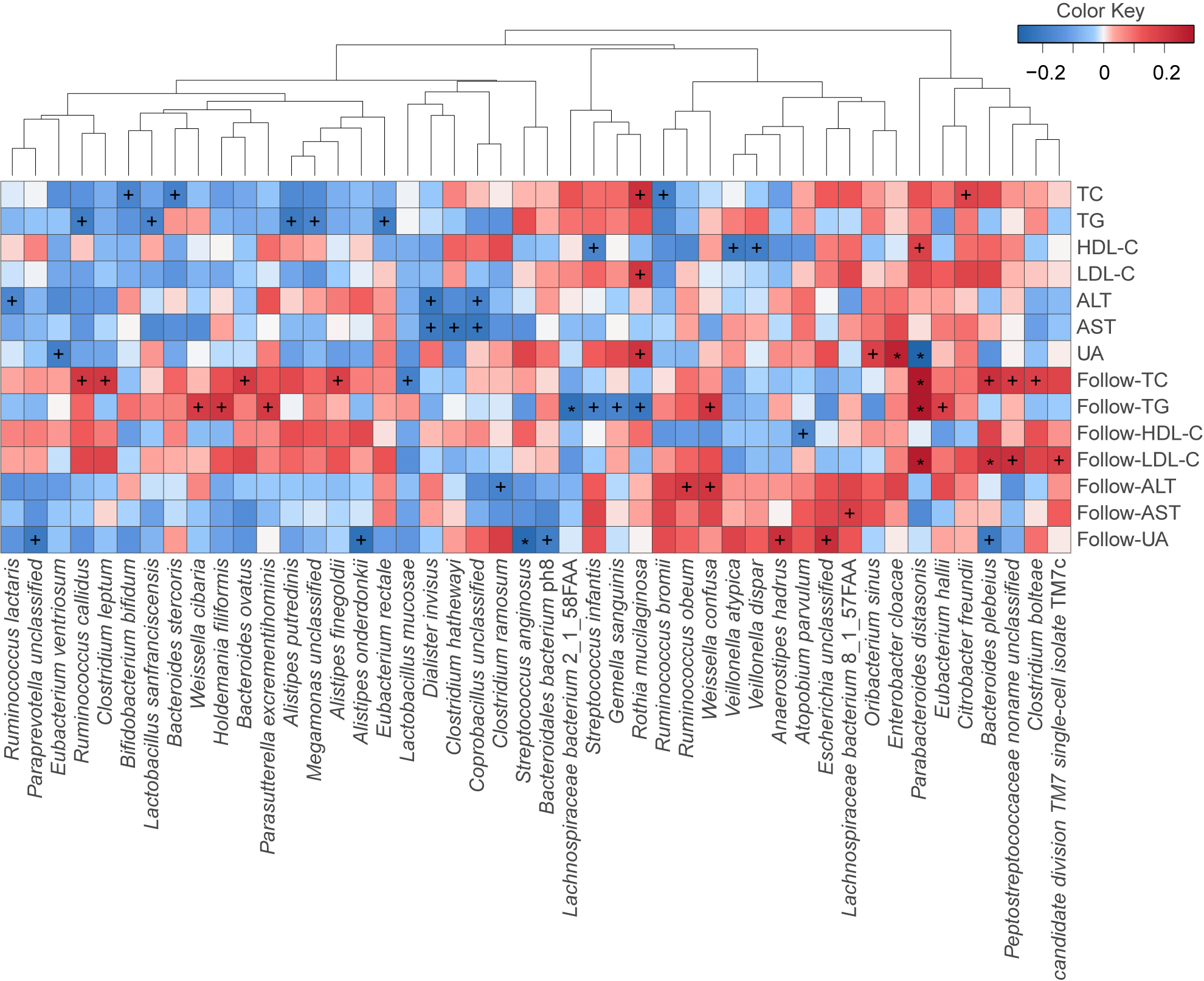

Supplement: Supplementary Figure S3 — Correlation between fecal microbiota and clinical indices (n = 126). Heatmap and hierarchical clustering of Spearman’s correlation coefficients between species and clinical indices at baseline and follow up. TC, total cholesterol, TG, triglyceride, HDL-C: high-density lipoprotein cholesterol; LDL-C: low-density lipoprotein cholesterol, UA, uric acid, AST, aspartate transaminase, ALT, alanine transaminase; +P < 0.05; *P < 0.01. [file Image_3.tif]
